# Recyclability of elastomer toughened recycled poly(ethylene terephthalate): The effect of grinding-extrusion-injection moulding on the mechanical and morphological properties of the blend

**DOI:** 10.1016/j.heliyon.2024.e32096

**Published:** 2024-05-29

**Authors:** Emese Slezák, Ferenc Ronkay, Dávid Réz, Katalin Bocz

**Affiliations:** a Department of Organic Chemistry and Technology, Faculty of Chemical Technology and Biotechnology, Budapest University of Technology and Economics, Műegyetem rkp. 3., H-1111, Budapest, Hungary; b Department of Innovative Vehicles and Materials, GAMF Faculty of Engineering and Computer Science, John von Neumann University, H-6000, Kecskemét, Hungary; c Department of Polymer Engineering, Faculty of Mechanical Engineering, Budapest University of Technology and Economics, Műegyetem rkp. 3., H-1111, Budapest, Hungary

**Keywords:** poly(ethylene terephthalate), Elastomer, Mechanical recycling, Reactive extrusion, Morphology, Mechanical properties

## Abstract

Reprocessing potential of recycled poly(ethylene-terephthalate) (RPET)/ethylene-butyl-acrylate-glycidyl methacrylate (EBA-GMA) blends was investigated. PET flakes from food packaging were compounded with 0, 5, 10, 15 and 20 % EBA-GMA. Injection moulded specimens were produced, and some of the specimens were grinded, and reproduced to simulate real reprocessing. It was revealed by scanning electron microscopy that the distribution and particle size of the elastomer did not change notably after recycling. Noticeable degradation of the polymer matrix was observable after every processing step. Such deterioration of PET resulted in higher crystallinity and rigid amorphous fraction, as found by differential scanning calorimetry, which ultimately led to higher storage modulus, while the notched impact strength and elongation at break decreased. Nevertheless, the mechanical performance of the reprocessed RPET/EBA-GMA blends still significantly outperforms that of the neat PET recyclate. The degree of chain breakage was found to be the primary factor determining the recyclability of RPET/EBA-GMA blends.

## 1 Introduction

Poly (ethylene terephthalate) (PET) is one of the thermoplastics applied in the greatest volume thanks to its favourable properties, including low gas permeability, inertness and sufficient stiffness [[1], [2], [3], [4], [5]]. Polymer recycling is an increasingly important topic, especially when it comes to the management of plastic packaging [6,7]. As for PET, several methods are available, from energy recovery [8] to chemical [9] and mechanical recycling. The latter is the conversion of purified polymer into granules/products by melt extrusion [10]. The main issue during the mechanical recycling of PET is the decaying mechanical properties (e.g. impact resistance, elongation at break), which is caused by high temperature, shear forces and the undergoing chemical reactions between the polymer and water/impurities. The combination of hydrolytic and thermo-oxidative degradation results in chain cleavage, which leads to the reduction of molecular weight [11].

Mechanical recycling of pure PET is often examined, and the recycled material can be successfully processed with various types of additives. Applying flame retardants to obtain upgraded RPET materials is a promising field. Ronkay et al. [12] managed to manufacture television parts from RPET/montmorillonite (MMT) composites, but the degradation of the recycled matrix was significant. To tackle this issue chain extender (CE) was added alongside the flame retardant agent and MMT and the thus improved rheological properties made the recycled material suitable for foaming applications [13,14]. To compensate for the chain-scission related loss in mechanical properties, elastomers are often blended to the recycled polymer. Kelnar et al. [15] added clay compatibilized rubber to RPET and reached a higher modulus due to the structural refinement of the components. RPET is also a suitable material for making reinforced composites. Ronkay et al. [16] compared the effect of glass fibres (GF) and basalt fibres and found that the latter causes smaller degradation of other properties (e.g. impact strength) in opposition to GF. Monti et al. [17] balanced the mechanical properties of RPET/GF composites by adding ethylene copolymers.

The consecutive recycling of PET was also examined by Mancini et al. [18]. The crystallinity increased with the number of recycling steps, and the chain breakage was proved by the elevated number of functional groups. Itim et al. [19] studied the effect of multiple extrusions on RPET and polypropylene (PP) contaminated RPET samples, and concluded that PP decreased crystallinity with increasing processing cycles due to the cross-linking reactions that reduced the chain mobility of PET. Furthermore, Kets et al. [20] simulated 5 mechanical recycling processes by consecutive extrusion on PP/PET blends compatibilized by poly(styrene-*co*-(ethylene-butylene)- styrene) grafted with maleic anhydride (SEBS-g-MAH). The reprocessing did not influence Young's modulus and tensile strength greatly, but the strain at break decreased when compatibilizer was not added. It was revealed that SEBS-g-MAH provided proper distribution of the PET particles, hence the tensile properties were adequate even after multiple extrusions. Nevertheless, the recycling of complex systems (blends, composites) needs to be further analysed as not many resources are available, especially on the mechanical recycling of PET/elastomer blends.

Ethylene-butyl-acrylate-glycidyl methacrylate (EBA-GMA) is a reactive compatibilizer and toughening agent applied for polyesters. At elevated temperatures, the epoxy group of the elastomer is capable of reacting with the PET chain ends [21]. You et al. administered EBA-GMA to PET/PLA blends, and it provided a 292 % increase in notched Izod impact strength with 14 % elastomer content, while the tensile modulus decreased by 25 % [22]. In a previous study carried out by Bocz et al. [23] it was revealed that toughening is more efficient with EBA-GMA when the matrix is RPET than when it is made of original PET (OPET). This is due to the higher concentration of reactive functional groups (hydroxyl, carboxyl) which result from the shorter, more deteriorated polymer chains. The Izod impact resistance values sharply increased above 10 % EBA-GMA content in the case of RPET, while a similar improvement was only noticed at 20 % elastomer ratio for OPET. Based on this observation, OPET/RPET/EBA-GMA blends were manufactured, and the impact resistance was significantly higher while the elastomer content could be decreased by 50 %. The degraded RPET molecules formed a toughening enhancer interface (TEI) and provided better compatibilization. The importance of TEI in PET/elastomer blends was further investigated by Ronkay et al. [24]. The study revealed that optimizing the moisture content of PET before reactive extrusion can promote the hydrolytic degradation of the polymer, thus highly reactive interphase can be created during processing. Consequently, a 6-fold improvement was achieved in impact strength just by eliminating the drying of PET. The influence of thermal annealing on RPET/EBA-GMA blends was also studied by Ronkay et al. [25]. The samples were heat treated at 150 °C for 0-20-40-60-180 s and the morphology was analysed in accordance with the three-phase model. Increasing EBA-GMA content led to higher ratios of rigid amorphous phase (RAF) for the untreated samples. However, during the annealing, the relaxation of RAF to mobile amorphous phase (MAF) and crystallization collectively influenced the quantity of the three phases and indirectly the mechanical properties. The former resulted in slightly higher impact strength values at shorter (20–40 s) annealing times.

The aim of the research is to reprocess recycled PET with EBA-GMA and examine the morphological and mechanical changes caused by mechanical recycling. The central question is whether further deterioration of the material by consecutive recycling makes it even more suitable for toughening, or whether the embrittlement induced by the lower molecular weight and enhanced crystallization will result in the reduction of impact properties.

## 2 Materials and methods

### 2.1 Materials

The recycled PET flakes were purchased from JP Pack Kft. (Hungary), which provides industrial quality raw material from food packaging with an intrinsic viscosity of 0.75 g/dl (e.g. bottles). The toughening agent was ethylene-butyl-acrylate-glycidyl methacrylate, commercially available as Elvaloy PTW (DuPont, USA). The additive contains 66.75 wt% ethylene, 28.00 wt% butyl-acrylate and 5.25 wt% glycidyl methacrylate. The elastomer has a low T_g_ (−55 °C) and melting temperature (72 °C) [26].

### 2.2 Methods

#### 2.2.1 Sample preparation

**Fig. 1:**
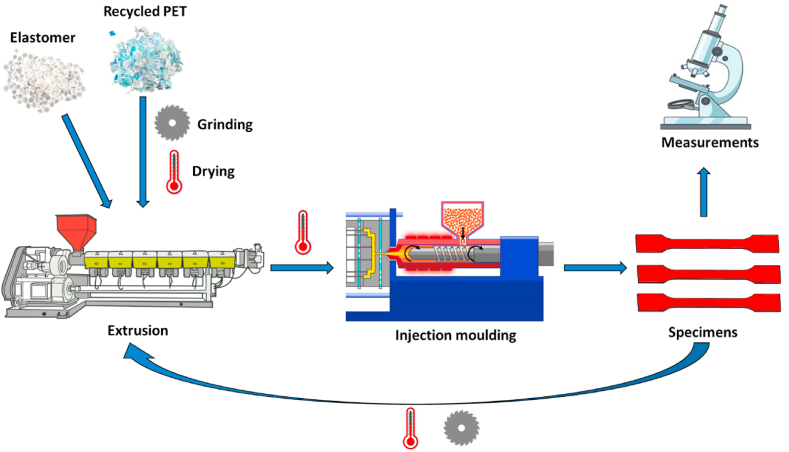
Scheme of the recycling process.

Prior to processing, the RPET flakes were further grinded on an SM 300 mill (Retsch, Germany), to facilitate feeding during extrusion. Then, the polymer was dried for 4 h in an UF1060 (Memmert, Germany) hot air dryer at 160 °C, to reduce its moisture content. An hour before processing the drying temperature was lowered to 90 °C to prevent the melting of EBA-GMA in the hopper. The elastomer was added in 0, 5, 10, 15 and 20 wt% ratios to the polymer. The reactive extrusion was performed on an LTE 26–48 (Labtech Scientific, Italy) twin-screw extruder. The screw speed was 80 rpm, the zone temperatures of the barrel were 255–265 °C, and the dye pressure was 37–52 bars. The extruded granules were dried again for 4 h at 160 °C, and then ISO dumbbell specimens were injection moulded on an ES 200/45 HL – V hydraulic machine (Engel, Austria). The barrel temperatures changed from 270 to 285 °C, the screw speed was 480 rpm, the holding pressure was 70–75 bars, the dye temperature was set to 45–55 °C and the cooling time lasted for 20–27 s. To test the recyclability of the blends, the same process was carried out one more time. The scheme of the recycling process is presented in Fig. 1.

**Fig. 2:**
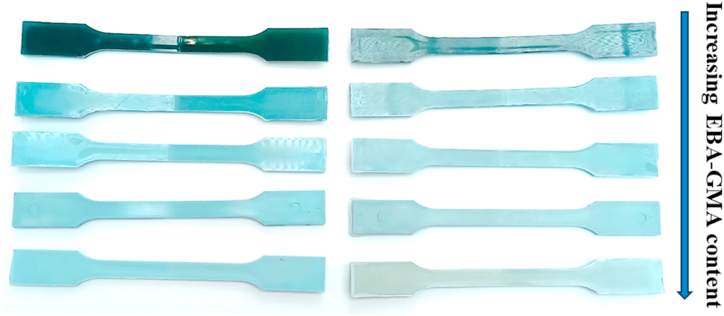
Photos of the injection moulded test specimens: 1x processed neat RPET (left) and 2x processed RPET with 0-5-10-15-20 % EBA-GMA content from top to bottom (right).

The photos of the injection moulded specimens can be seen in Fig. 2. It is observable that the samples produced from blue mineral water bottles became lighter and less glossy with increasing EBA-GMA content. After the 2^nd^ processing cycle, the color of the specimens turned greener, this can be explained by the yellowing of the blue base color caused by degradation.

#### 2.2.2 Characterisation methods

The intrinsic viscosity (IV) of the bottle flakes, the granules and the injection moulded specimens that do not contain any EBA-GMA were measured on an RPV-1 (PSL Rheotek, UK) viscosimeter. The sample was dissolved in 60:40 % phenol – 1,1,2,2-tetrachloro-ethane and the concentration of the tested solution was 0.5 g/dl. The test was carried out at 30 °C.

Izod impact test with a 5115 10/01 (Zwick, Germany) impact tester was carried out on the specimens cut from the injection moulded products. A 2 mm deep, V-shaped notch was applied on all specimens with an RM2125 RT (Leica, Germany) rotation microtome. 10 parallel measurements were performed. The energy of the pendulum was 5.5 J and the energy detected when no specimen was in its way was 0.033 J.

Tensile test with 5 parallel measurements was performed on the injection moulded specimens with a 3369 (Instron, USA) universal mechanical tester. The clamping distance was set to 115 mm and the tensile rate was 50 mm/min. The measurements lasted until the specimens broke.

The dynamic mechanical analysis (DMA) was done in tensile arrangement on a DMA25 (MetraVIB, France). 30 x 8 x 2 mm specimens were prepared, 2 from each composition. The temperature increased from 10 to 140 °C with a rate of 3 °C/min, and the frequency of deformation was 10 Hz. The glass transition temperature (T_g_ [°C]) was determined by the maximum of the tan δ peak.

A DSC 131 Evo (Setaram, France) differential scanning calorimeter (DSC) was used to determine the ratio of crystalline, mobile, and rigid amorphous phases, as well as to acquire the melting temperature. The specimen was tested in nitrogen and the heating/cooling rate was 20 K/min. First, the specimen was heated from 20 to 320 °C, kept at 320 °C for 5 min, then cooled to 20 °C and remained at 20 °C for 5 min, then heated to 320 °C again. The ratio of crystallinity was calculated with eq. (1):

(1) $$\chi_{c\;}\left[\%\right]=\frac{{\Delta H}_{m}-{\Delta H}_{cc}}{\Delta {H_{m}}^{0}\left(1-\varphi_{EBA-GMA}\right)}∙100\%$$

where χ_c_ is the crystalline ratio of the sample [%], ΔH_m_ is the crystalline melting enthalpy [J/g], ΔH_cc_ is the enthalpy of cold crystallization [J/g], ΔH_m_^0^ is the melting enthalpy of a perfect PET crystal (140 [J/g]) and φ_EBA-GMA_ is the mass ratio of the elastomer [−].

The relative size of the mobile amorphous phase was determined in accordance with eq. (2):

(2) $$\chi_{m\;}\left[\%\right]=\frac{{\Delta c}_{p}}{\Delta {c_{p}}^{0}\left(1-\varphi_{EBA-GMA}\right)}∙100\%$$

where χ_m_ is the ratio of the mobile amorphous phase [%], Δc_p_ is the specific heat change during glass transition [J/(g·K)] and Δc_p_^0^ is the specific heat change of a fully amorphous PET sample (0.405 [J/(g·K)]).

The ratio of the rigid amorphous phase was calculated with eq (3):

(3) $$\chi_{r\;}\left[\%\right]=100\%-\chi_{c\;}-\chi_{m\;}$$

where χ_r_ is the ratio of the rigid amorphous phase [%].

The surface structure of the impact-tested specimens was examined by an EVO MA 10 (ZEISS, Germany) type scanning electron microscope (SEM) and the magnification was 500x. Additionally, injection moulded test specimens were embedded in acrylic resin and the surface was polished with an LS Twin (LSA Remet, Australia) polisher until 1 μm surface roughness was reached, then the elastomer phase was dissolved in toluene for 2 h at room temperature, and the residual solvent was removed in a V0500 vacuum drying oven (Memmert, Germany) at 60 °C and 90 mbar, overnight. Each sample was coated with a gold layer by a Q 150R S gold coater (Quorum, England). The accelerating voltage was set to 15.00 kV, and the magnifications were 500x and 5000x. The working distance was between 7.5 and 9.5 mm.

## 3 Results and discussions

### 3.1 Degradation of PET during melt-processing

**Fig. 3:**
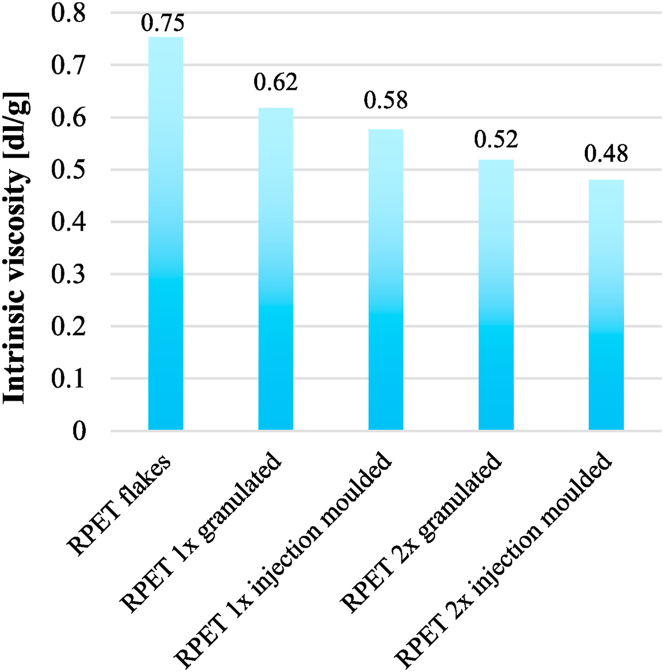
Intrinsic viscosity of 100 % RPET after the different processing steps.

To investigate the degradation, the intrinsic viscosity (IV) of neat RPET was tested after each stage of processing. Due to the insolubility of EBA-GMA in phenol-1,1,2,2-tetrachloro-ethane, only the samples without elastomer were examined. However, as the processing parameters were similar, it can be assumed that the degradation of PET in elastomer toughened blends is comparable to what was observed in the case of neat RPET samples. As the polymer was exposed to high temperature and shear stress multiple times, the polymer chains underwent cleavage, indicated by the decrease of intrinsic viscosity (IV) (Fig. 3). After the second cycle, the IV reduced below 0.50 dl/g, which indicates significant degradation. Depending on the application of the product, the molecular weight and IV of original PET can vary in a wide range, for example, the intrinsic viscosity of bottle-grade PET is usually 0.74–0.82 dl/g [27], while the molecular weight ranges from 24,000 to 36,000 g/mol [28].

**Fig. 4:**
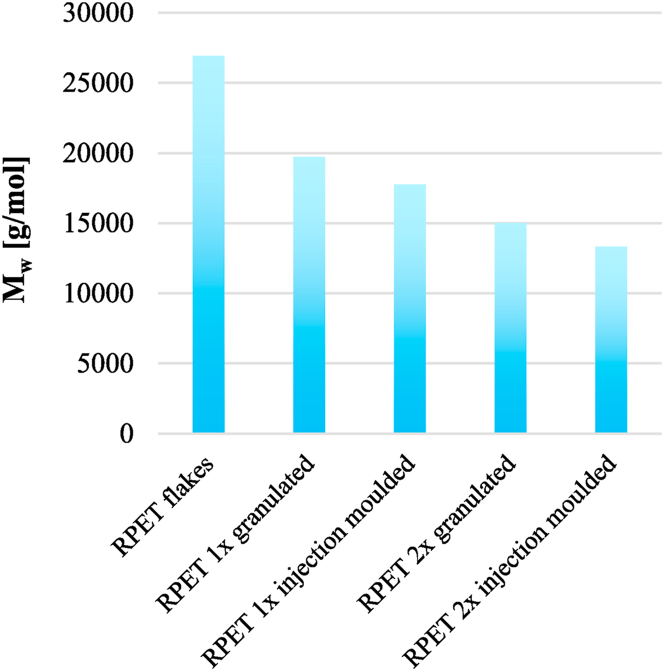
Weight average molecular weight at different recycling stages.

Based on a previous study carried out by Ronkay et al. [29] the molecular weights of PET samples with different IV values were measured by gel permeation chromatography (GPC) and by fitting the Mark-Houwink equation to these points (**S 1**), the weight average molecular weight (M_w_) for our samples can be calculated, as well (Fig. 4). The following equation was applied for the determination of the molecular mass:

(4) $$IV=1.10∙10^{-3}∙M_{w}^{0.64}$$

Each processing step led to the reduction of the molar mass and after the 2^nd^ injection moulding, the weight average molecular weight dropped by approximately 50 % compared to the flakes. Below a critical mass, molecular entanglements cannot be formed, which can cause a remarkable drop in impact resistance [23]. Oreski et al. [5] reported a M_w_ of 14.000–15.000 g/mol as the critical value of ductile/brittle behaviour, and after the 2^nd^ injection moulding the pure RPET samples approach this minimum value, therefore brittle fracture can be assumed in the case of the additive-free material.

### 3.2 Change of mechanical properties with recycling

**Fig. 5:**
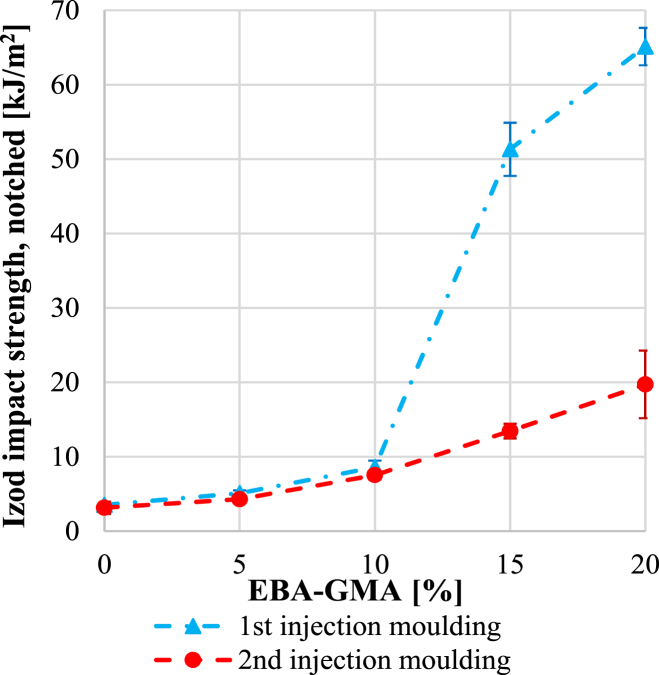
Izod impact strength after the 1^st^ and 2^nd^ recycling.

Fig. 5 presents the impact strength values after the 1^st^ and 2^nd^ injection moulding. The 1^st^ injection moulded samples are in good agreement with our previous studies [25]. However, after the second cycle, the results fall short, even at higher elastomer ratios, though an increment of impact strength is observable with increasing elastomer content. The effect of recycling on impact strength can be caused by two phenomena. The first is the degradation of the PET matrix, while the second effect is the possible change of the dispersion of elastomer during reprocessing. The degradation of the PET matrix is certainly an issue based on Fig. 3, where it can be seen that after the 2^nd^ processing cycle, the IV of PET reduced from 0.58 dl/g to 0.48 dl/g (by 17 %). It is proved, that after a certain degree of IV reduction, the impact strength significantly drops. According to the experimental results of Oromiehie and Mamizadeh [30], the reduction in the Izod impact strength of pure PET can reach 20 % at a 0.1 dl/g IV drop. Our recent study revealed [24] that at 13 % EBA-GMA content, if the IV of the matrix polymer decreases from 0.5 dl/g to 0.4 dl/g (by 20 %), then the impact resistance reduces by 85 %, thus the toughening effect of EBA-GMA in such a low range of IV is minimal.

**Fig. 6:**
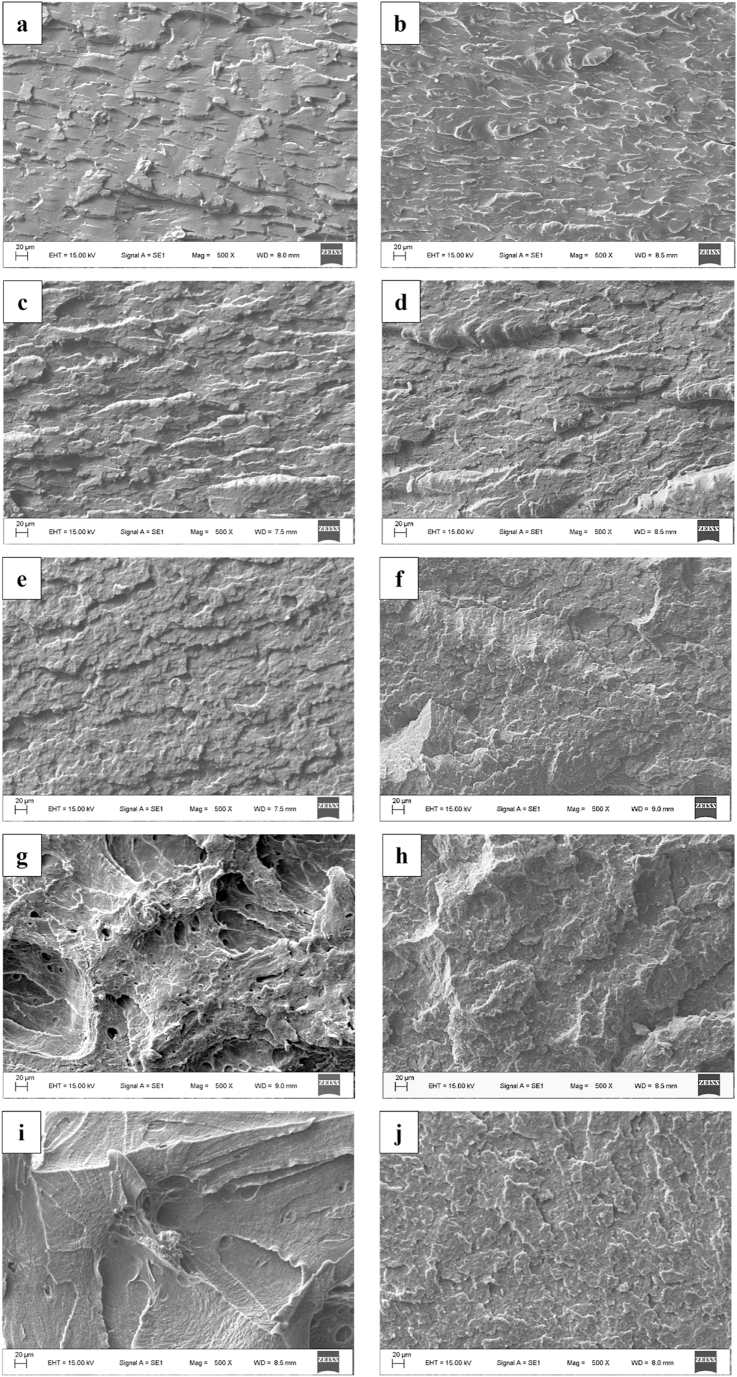
SEM image of fracture surfaces: a) 0 % EBA-GMA 1x inj. moulded; b) 0 % EBA-GMA 2x inj. moulded; c) 5 % EBA-GMA, 1x; d) 5 % EBA-GMA, 2x; e) 10 % EBA-GMA, 1x; f) 10 % EBA-GMA, 2x; g) 15 % EBA-GMA, 1x; h) 15 % EBA-GMA, 2x; i) 20 % EBA-GMA, 1x; j) 20 % EBA-GMA, 2x

To find the reason behind the deteriorated impact strength values, the fracture surfaces were studied by scanning electron microscope; the micrographs are presented in Fig. 6. The images confirmed that the reactive toughening was only efficient after the first processing cycle at 15 and 20 % EBA-GMA, where the fracture is ductile, while all the other samples suffered brittle fracture.

**Fig. 7:**
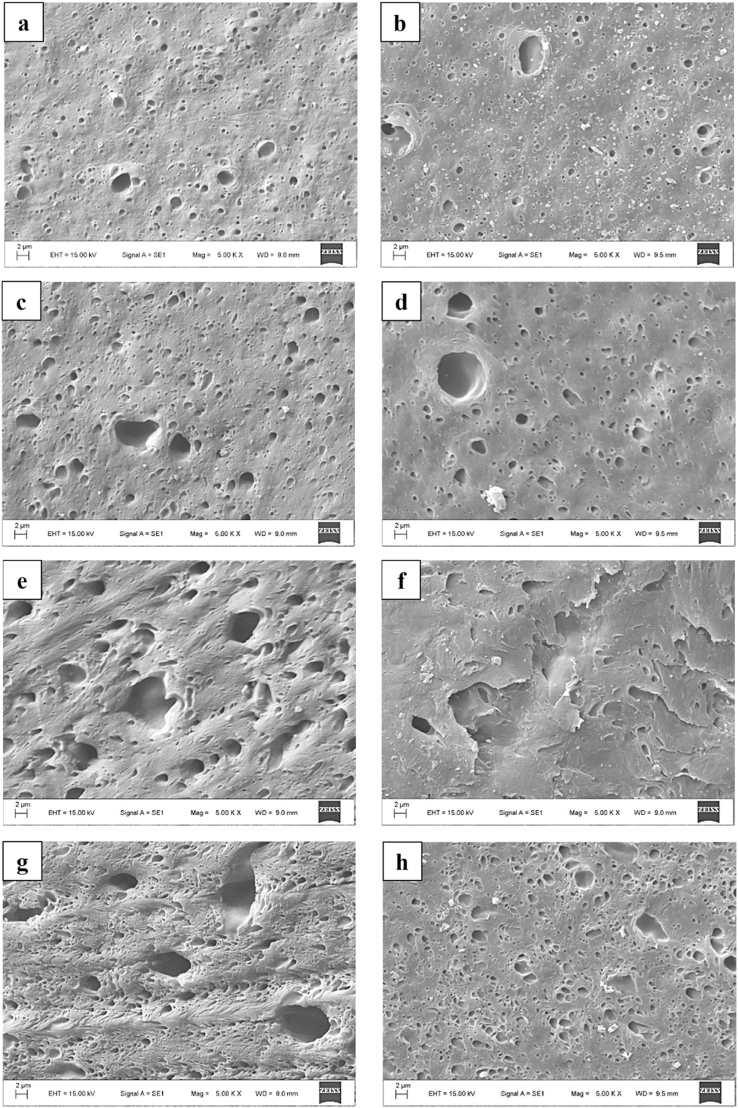
*SEM micrographs on samples:* a) *5 % EBA-GMA, 1x injection moulded; b) 5 % EBA-GMA, 2x inj. moulded; c) 10 % EBA-GMA, 1x; d) 10 % EBA-GMA, 2x; e) 15 % EBA-GMA, 1x; f) 15 % EBA-GMA, 2x; g) 20 % EBA-GMA, 1x; h) 20 % EBA-GMA, 2x*.

In order to make the dispersion of elastomer particles visible, EBA-GMA was selectively dissolved in toluene and the size of the voids left behind was examined. The SEM images are presented in Fig. 7.

**Fig. 8:**
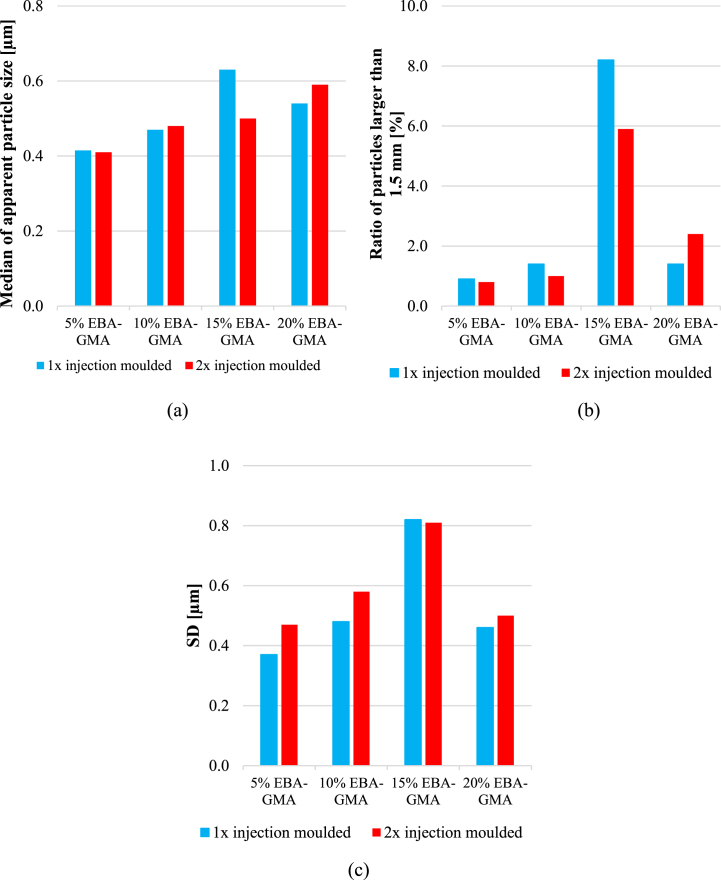
a) The median of apparent particle size, b) the ratio of particles bigger than 1.5 μm and c) the standard deviation of particle size at different EBA-GMA contents.

To quantify the possible difference between the images, the apparent particle sizes were measured manually with AxioVision SE64 Rel, 4.9.1 software and the median values of approximately 400–700 particles were calculated and depicted in Fig. 8***/a***. The median values were below 1 μm in all cases, indicating a good distribution of EBA-GMA within the RPET matrix. Seemingly, the consecutive extrusion did not noticeably affect the dispersion, even though it can be presumed that during the 2^nd^ recycling, the decreased intrinsic viscosity would result in bigger particle size while the shear forces within the processing machines might reduce the particle size. Thus, multiple – opposing – effects are present at the same time. In this case, such growth or decrease in size was not noticed – or only minimally –, probably due to the covalent bonds between PET and EBA-GMA and the partial cross-linking of elastomer particles which hinders their complete melting [24,25,31]. With increasing EBA-GMA content the particle size was slightly increasing. At 15 % EBA-GMA content, the median particle size of the once injection moulded samples was a little higher, exceeding the values measured for 20 % EBA-GMA content. This can be explained by particle size distribution characteristics, namely a lot of small and a few bigger particles were detected. The ratio of particles bigger than 1.5 mm varied but did not exceed 10 % in any case. It is probable that smaller particles are more prone to cross-link (due to their higher specific area more chemical reactions can start on the interface), thus the shape/size of these smaller elastomer particles only slightly changes during the second processing. However, the size of the larger particles can still vary, they can break up or even coalesce depending on the viscosity ratio of the ingredients and shear forces [32]. The smaller, partially cross-linked EBA-GMA particles dispersed in the PET melt increase the apparent viscosity of the melt, which can result in higher shear forces. At 15 % EBA-GMA content, the proportion of large particles increased significantly: from 0.8-1.6 % to 6–8%, then at 20 % EBA-GMA content, a decrease is visible again (Fig. 8***/b***). The particle size enhancing effect of the increasing EBA-GMA content and the particle size reducing effect of apparent melt viscosity increase due to the increasing number of partially cross-linked small-sized particles are thus reversed between 15 and 20 % EBA-GMA content. This is also indicated by the fact that the standard deviation of the distribution is the largest for this composition (Fig. 8***/c***). It should be noted that the median value of the entire distribution is only slightly changed by these effects, and the impact strength diagram (Fig. 5) does not show any significant effect of this difference in particle size distribution between 15 and 20 % EBA-GMA content.

**Fig. 9:**
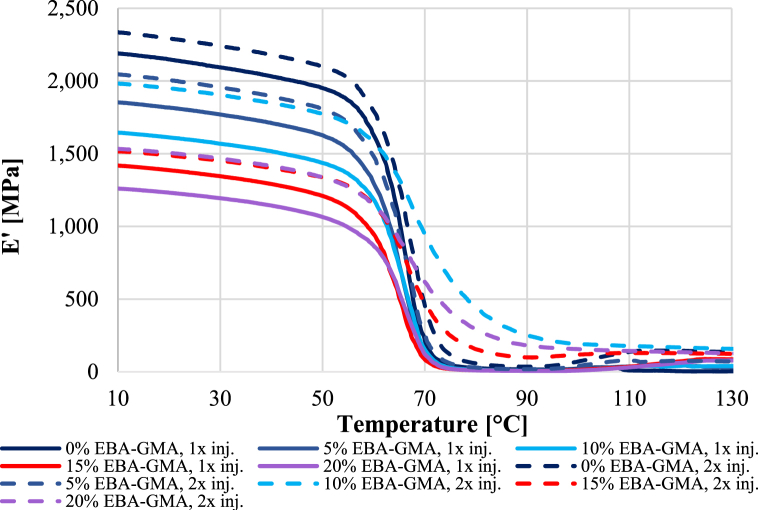
Temperature dependence of storage moduli.

**Fig. 10:**
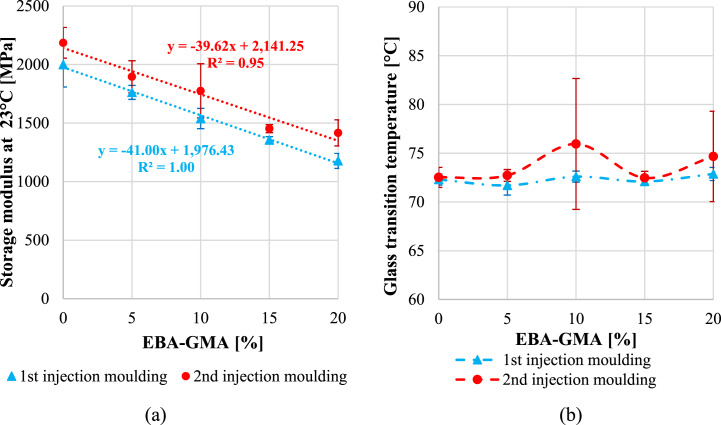
a) Room temperature storage moduli and b) glass transition temperature after the 1^st^ and 2^nd^ recycling.

Apart from impact resistance, the dynamic mechanical properties were also examined. A DMA curve of each sample is presented in Fig. 9. The storage moduli were increasing with less EBA-GMA content and more processing cycles. The latter can be caused by the higher crystallinity of the reprocessed samples. It is also visible, that around the glass transition the moduli sharply dropped in the case of the 1^st^ cycle, while the samples from the 2^nd^ cycle showed better heat resistance. In Fig. 10***/******a***, the room temperature storage moduli (at 23 °C) were highlighted. The elastomer content dependence showed a good linear correlation in the examined additive range. Observing Fig. 10***/b,*** the glass transition temperatures were similar, regardless of the reprocessing and the elastomer content.

**Fig. 11:**
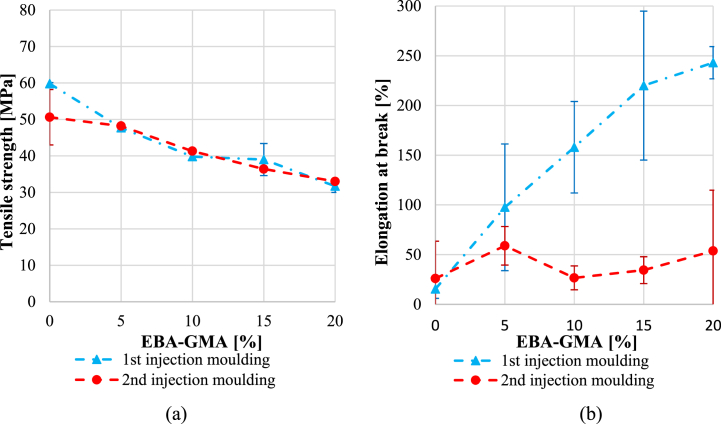
a) Tensile strength and b) elongation at break after the 1^st^ and 2^nd^ recycling.

The tensile properties were quantified, as well, however, Fig. 11***/a*** suggests that the difference caused by recycling in tensile strength was negligible. This is similar to what Kets et al. reported in their study [20]. In contrast, elongation at break (Fig. 11/b) was significantly higher after the 1^st^ cycle and sharply increased with the elastomer content, while as a result of reprocessing the values dropped, and the EBA-GMA content did not influence the results substantially. For polymers, the elongation at break usually shows a similar trend to the impact strength [33,34] since both properties are remarkably dependent on the average molecular weight. In the case of the once-processed samples, the 0.58 dl/g IV value was still satisfactory for expressing the toughening effect of EBA-GMA, while for the twice processed samples, the 0.48 dl/g IV value was not sufficient for this.

### 3.3 Crystalline properties

**Fig. 12:**
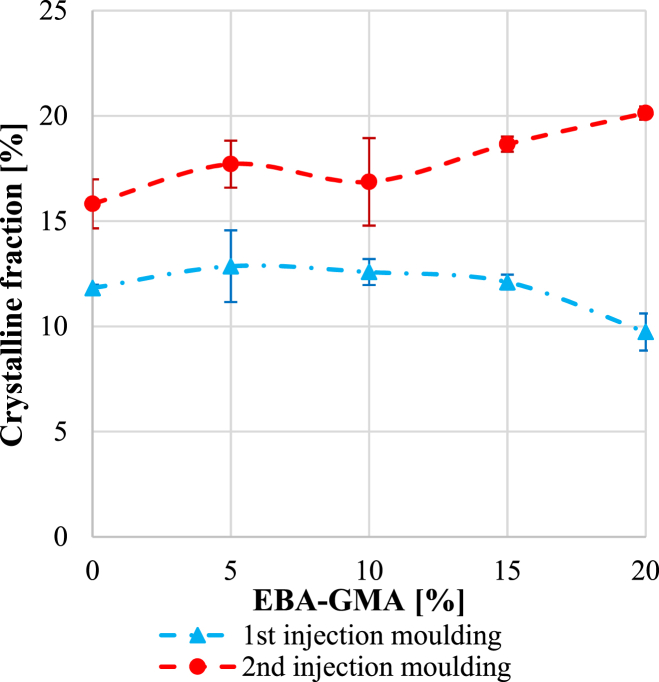
Crystallinity after the 1^st^ and 2^nd^ injection moulding (as determined from the 1^st^ heating curve).

As the dispersion of the elastomer particles did not change greatly (Fig. 7, Fig. 8), the morphology of the samples was analysed by DSC to see the influence of the degradation. The ratios of the 3 fractions were determined from the DSC curves, based on equations (1), (2), (3) (see section 2.2.2). The crystallinity was about 10–13 % after the first cycle, while 15–20 % after the second (Fig. 12), which explains the differences in the DMA results (storage modulus) (Fig. 9). The difference is caused by the decreasing viscosity (Fig. 3, Fig. 4) and chain length, which facilitate the rearrangement of polymer chains [30]. After the 1st processing cycle, the crystallinity was slightly decreasing with the elastomer content, but after the reprocessing just the opposite was experienced. The decreasing crystallinity indicates a strong bond between PET and EBA-GMA, which results in reduced polymer chain mobility [19]. However, the chain cleavage induced by reprocessing enhanced the chain mobility and presumably no new connection points were formed between PET and EBA-GMA, thus crystallization is not hindered anymore by the elastomer.

**Fig. 13:**
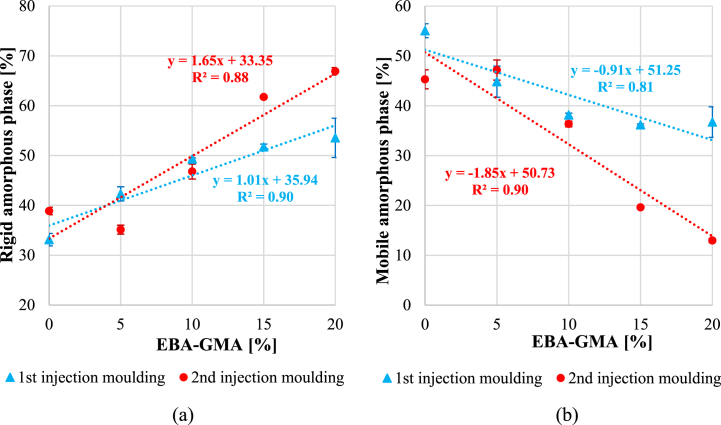
a) Rigid amorphous fraction and b) mobile amorphous phase after the 1^st^ and 2^nd^ injection moulding (as determined from the 1^st^ heating curve).

Fig. 13 presents the rigid (a) and mobile (b) amorphous phases. It was already revealed in our previous study [25], that the higher the elastomer content, the higher the rigid amorphous phase (RAF) is, and Fig. 13/***a*** confirms it, as well. The reprocessing also increased RAF which is in connection with the higher crystallinity of the samples. Fig. 13***/b*** shows MAF with increasing EBA-GMA content and a linear correlation can be observed. The 2^nd^ processing cycle resulted in a great drop in the mobile phase, which coupled with the higher ordered fractions can explain the weaker impact properties.

## 4 Conclusions

Within the topic of polymer blend recycling, our study aimed to give information on the potential applicability and limitations of using recycled PET materials. Industrial recycling process of RPET/EBA-GMA blends with different elastomer contents was simulated by carrying out two cycles of grinding – extrusion – injection moulding. Our results showed that the impact strength of the blends noticeably decreased in the 2^nd^ cycle, especially at higher elastomer ratios (15 and 20 %), whereas the toughening effect was exceptionally good in the 1^st^ cycle. Apart from impact strength and elongation at break, other mechanical properties (e.g. storage modulus, tensile strength) only marginally changed.

Based on morphological analyses, it was concluded that the size distribution and median of the apparent particle size of the elastomer phase barely changed during reprocessing cycles. This is likely due to the reactive compatibilization of the components and the cross-linking reactions in the EBA-GMA particles, which stabilize the phase structure even after the first processing. In parallel to the observed quasi-stabilized elastomer phase and blend morphology, a significant increase in the degradation state of the PET phase was evinced during each reprocessing step. The degradation of the matrix polymer was identified as the main cause of the reduction in impact strength of the blends. Namely, the shortening of the molecular chains during each processing step resulted in an increase in the ratio of the ordered phase (CRF + RAF), which is associated with a decrease in toughness. Also, above a certain degree of degradation, the insufficient entanglement density can result in a sharp drop in the impact resistance, and this critical range of molecular weight of RPET was approached after the 2^nd^ processing cycle. Altogether, even after a full reprocessing cycle of extrusion-grinding-injection moulding, the RPET/EBA-GMA blends were found to have great stiffness and sufficient impact strength, which makes them suitable to produce technical parts, household items and containers.

It has to be noted, that since these days the sorting and separation of polymer blends is not solved or rather problematic [35], the polymer blends entering the recycling stream are considered to contaminate the recyclable materials. The information on the recyclability of PET blends presented in this work is therefore primarily relevant and forward-looking from the point of view of the in-house waste management of blend manufacturers. Since the degree of chain breakage was found to be the primary factor determining recyclability, infinite recycling is probably not possible for recycled PET-based blends. However, if mixing with virgin materials can be considered, it is likely that a high proportion of recycled content can be achieved in PET-based blends and in the products made thereof without any technical concerns. Increasing the recycled contents in plastic products is further encouraged in addition to the increasingly strict legal regulations [36], as well as the ever-increasing price of virgin polymer raw materials.

## Data availability

The data are available from the corresponding author upon reasonable request.

## CRediT authorship contribution statement

**Emese Slezák:** Writing – original draft, Visualization, Methodology, Investigation. **Ferenc Ronkay:** Writing – review & editing, Supervision, Methodology, Investigation, Conceptualization. **Dávid Réz:** Writing – original draft, Visualization, Methodology, Investigation. **Katalin Bocz:** Writing – review & editing, Supervision, Conceptualization.

## Declaration of competing interest

The authors declare that they have no known competing financial interests or personal relationships that could have appeared to influence the work reported in this paper.
